# Involvement of Caveolin-1 in Repair of DNA Damage through Both Homologous Recombination and Non-Homologous End Joining

**DOI:** 10.1371/journal.pone.0012055

**Published:** 2010-08-06

**Authors:** Hua Zhu, Jingyin Yue, Zui Pan, Hao Wu, Yan Cheng, Huimei Lu, Xingcong Ren, Ming Yao, Zhiyuan Shen, Jin-Ming Yang

**Affiliations:** 1 Department of Pharmacology and The Penn State Hershey Cancer Institute, The Pennsylvania State University College of Medicine, and Milton S. Hershey Medical Center, Hershey, Pennsylvania, United States of America; 2 Department of Pharmacology, Robert Wood Johnson Medical School, University of Medicine and Dentistry of New Jersey, New Brunswick, New Jersey, United States of America; 3 Department of Radiation Oncology, Robert Wood Johnson Medical School, University of Medicine and Dentistry of New Jersey, New Brunswick, New Jersey, United States of America; 4 Department of Physiology and Biophysics, Robert Wood Johnson Medical School, University of Medicine and Dentistry of New Jersey, New Brunswick, New Jersey, United States of America; National Institutes of Health, United States of America

## Abstract

**Background:**

Caveolin-1 (Cav-1), the major component of caveolae, is a 21–24 kDa integral membrane protein that interacts with a number of signaling molecules. By acting as a scaffolding protein, Cav-1 plays crucial roles in the regulation of various physiologic and patho-physiologic processes including oncogenic transformation and tumorigenesis, and tumor invasion and metastasis.

**Methodology/Principal Findings:**

In the present study we sought to explore the role of Cav-1 in response to DNA damage and the mechanism involved. We found that the level of Cav-1 was up-regulated rapidly in cells treated with ionizing radiation. The up-regulation of Cav-1 following DNA damage occurred only in cells expressing endogenous Cav-1, and was associated with the activation of DNA damage response pathways. Furthermore, we demonstrated that the expression of Cav-1 protected cells against DNA damage through modulating the activities of both the homologous recombination (HR) and non-homologous end joining (NHEJ) repair systems, as evidenced by the inhibitory effects of the Cav-1-targeted siRNA on cell survival, HR frequency, phosphorylation of DNA-dependent protein kinase (DNA-PK), and nuclear translocation of epidermal growth factor receptor (EGFR) following DNA damage, and by the stimulatory effect of the forced expression of Cav-1 on NHEJ frequency.

**Conclusion/Significance:**

Our results indicate that Cav-1 may play a critical role in sensing genotoxic stress and in orchestrating the response of cells to DNA damage through regulating the important molecules involved in maintaining genomic integrity.

## Introduction

Caveolin-1 (Cav-1), a major structural protein of caveolae, is involved in many physiologic and patho-physiologic processes such as cardiovascular diseases, neurological disorders, and cancers. Although accumulating evidence indicate that expression of Cav-1 is altered in a stage-dependent manner during progression of various types of cancers [1], [2], [3], [4], the precise roles of Cav-1 in cancer development, progression, and treatment remain to be fully defined. Based on its location at chromosome 7 (7q31.1), which is frequently deleted in human malignancies [5], Cav-1 is believed to be a tumor suppressor. Indeed, Cav-1 was found to be down-regulated in many types of cancers including breast cancer [6], colon cancer [7], lung cancer [8], [9], [10], ovarian cancer [11], [12], sarcomas [13], and thyroid cancer [14]. Forced expression of Cav-1 inhibits tumor growth and induces apoptosis of tumor cells [15], [16]. Additionally, a mutation in Cav-1 at codon 132 (P132L) was found in 16% of the primary human breast cancer cases [17], and interbreeding Cav1^−/−^ mice with MMTVPyMT mice (mouse mammary tumor virus-Polyoma middle T antigen) accelerated onset of mammary tumors in their offspring [18].

On the other hand, up-regulation of Cav-1 has been observed in highly metastatic human cancers, and is associated with poor clinical prognosis [10], [19], [20], [21], [22], [23], [24], [25], [26], [27], [28], [29], [30] and with resistance to therapy [31], [32]. These observations indicate that re-expression of Cav-1 at advanced stages of cancer may play a pro-survival role that protects tumor cells against various stresses such as micro-environmental and therapeutic stress. Recently, it was demonstrated that expression of Cav-1 promotes survival of cancer cells following treatment with ionizing radiation (IR) [33], [34], further supporting Cav-1 as a stress protector in malignant cells. The protective effect of Cav-1 on IR-treated cells also suggests that this signaling-modulating molecule may play an important role in repair of damaged DNA. The main DNA damage caused by IR is double strand break (DSB), which can be repaired by two major pathways: homologous recombination (HR) and non-homologous end joining (NHEJ). HR pathway can accurately repair DSB via exchange of genetic material between two similar or identical strands of DNA; NHEJ is a repairing process in which the break ends are directly ligated without the need for a homologous template and thus is error-prone. As damage of DNA not only causes neoplasm but is also utilized in therapeutic interventions such as radiotherapy and chemotherapy, and as Cav-1 is differentially expressed during tumor progression, understanding the role of Cav-1 in DNA DSB repair and the underlying mechanism(s) may help further decipher the signaling pathways involved in tumor initiation and progression, and help develop new approaches to the prevention and treatment of cancers. We report here that the up-regulation of Cav-1 protein in response to DNA damage plays an important role in activating DNA repair signaling cascade and in promoting repair of DSB through both HR and NHEJ, thus contributing to maintenance of genomic integrity.

## Results

### Genotoxic stress induces a transcriptionally independent up-regulation of Cav-1

**Figure 1:**
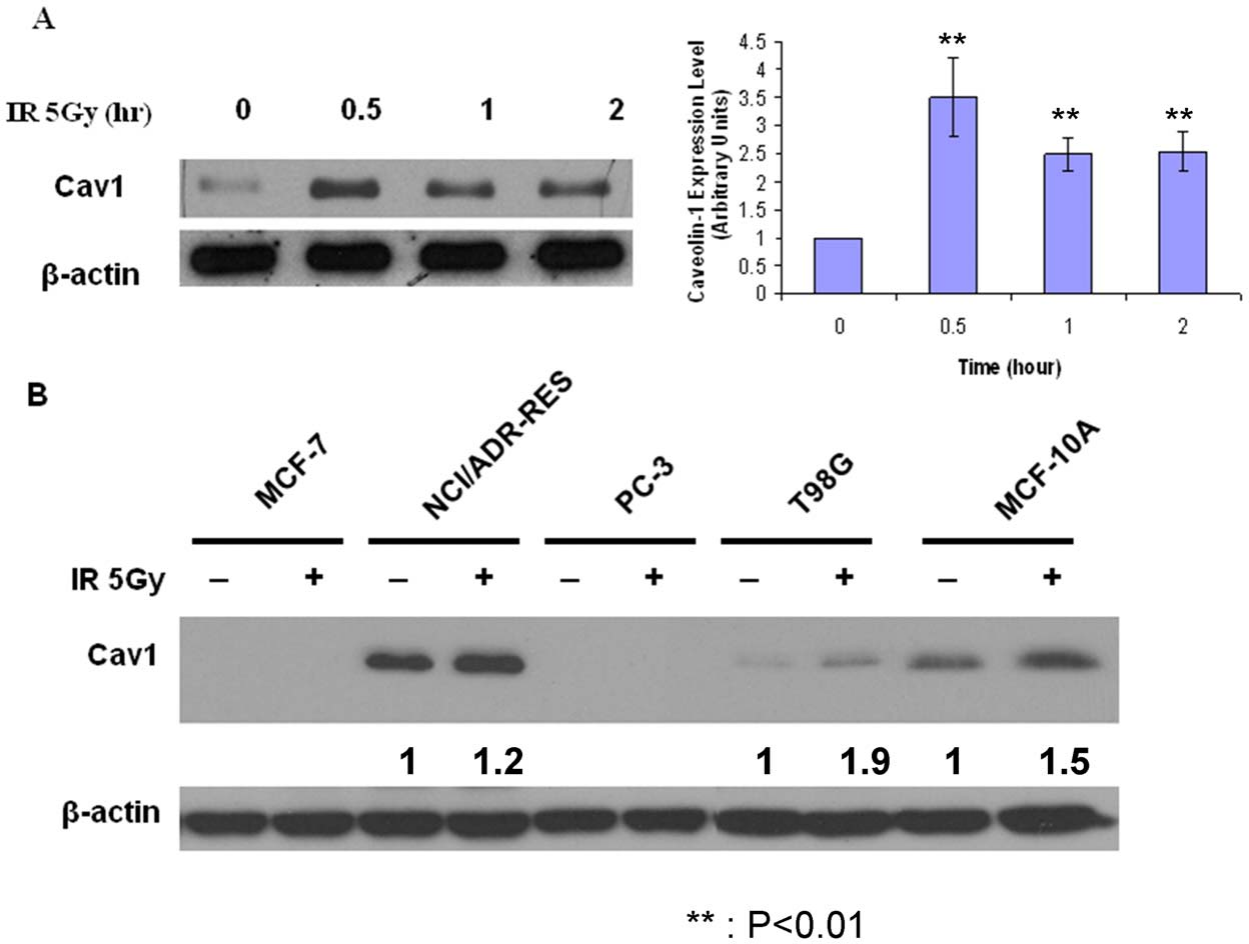
Treatment with IR stimulates the expression of Cav-1 protein. (**A**) MDA-MB-468 cells were irradiated (5 Gy) for the indicated period of time, and then the treated cells were collected for Western blot analysis of Cav-1. β-actin was used as a loading control. Expression of Cav-1 and β-actin were quantified using imageJ software, and Cav-1 level was normalized to that of β-actin. The normalized Cav-1 at the zero time point was arbitrarily set as 1. Bar represent mean ± S.D. of three separate experiments. (**B**) MCF-7, NCI/ADR-RES, PC-3, T98G and MCF-10A cells were treated or untreated with 5 Gy ionizing radiation, and Cav-1 expression was analyzed by Western blot. Results shown are the representative of three identical experiments.

**Figure 2:**
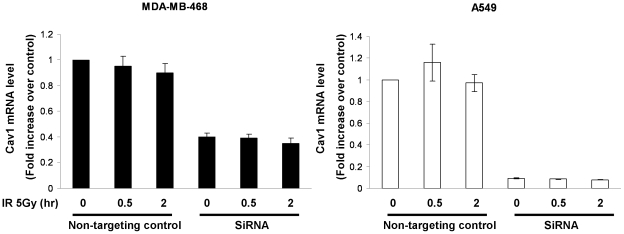
IR has no effect on expression of Cav-1 mRNA. MDA-MB-468 and A549 cells were transfected with a non-targeting RNA or siRNA against Cav-1. Twenty-four hours later, cells were treated with 5 Gy radiation for the indicated period of time. To determine Cav-1 mRNA, total RNAs were extracted from the cells and quantitative real-time RT-PCR was performed. Cav-1 mRNA level was normalized to β-actin mRNA. The Cav-1 mRNA level of the cells treated with the non-targeting RNA and without IR treatment was arbitrarily set as 1. Results shown are the representative of three similar experiments; each bar represents mean ± SD of quadruplicate determinations.

Expression of Cav-1 was reported to be elevated in cells exposed to IR [33], [34]. As shown in Fig. 1A, treatment with IR stimulated the expression of Cav-1 protein in MDA-MB-468 cells. The DNA damage-induced Cav-1 up-regulation also occurred in other cell lines (both tumor cells and non-tumor cells) expressing endogenous Cav-1 such as NCI/ADR-RES, T98G and MCF-10A, but not in cell lines (MCF-7 and PC-3) that do not express endogenous Cav-1 (Fig. 1B), and did not appear to result from altered transcription of the Cav-1 gene, because IR did not affect the level of Cav-1 mRNA in MDA-MB-468 and A549 cells with or without silencing of Cav-1, as determined by qRT-PCR (Fig. 2). With the use of these cell lines containing different status of p53, it appeared that IR – induced alteration of Cav-1 was independent of p53 status.

### Expression of Cav-1 is associated with DNA damage response pathways

**Figure 3:**
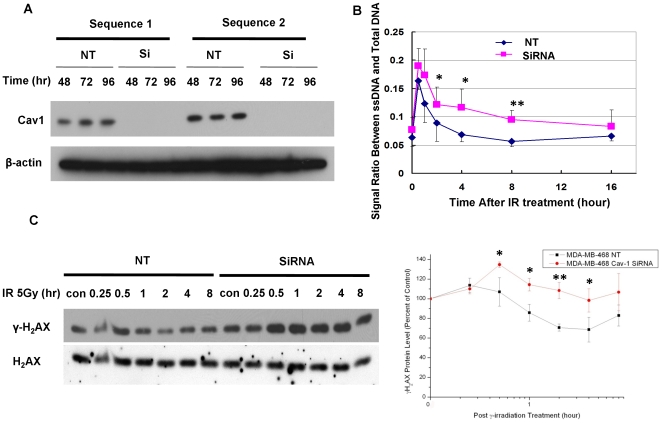
Silencing of Cav-1 expression by siRNA increases the IR-induced accumulation of ssDNA and γ-H2AX. (**A**) MDA-MB-468 cells were transfected with a non-targeting RNA (NT) or either Cav-1-targeted siRNA sequence 1 or sequence 2. At the indicated time following transfection, the cells were collected for Western blot analysis of Cav-1. β-actin was used as a loading control. (**B**) MDA-MB-468 cells were transfected with a Cav-1 siRNA or a non-targeting RNA, followed by IR (5 Gy). The cells were collected at the indicated time points and fixed for immunofluorescent detection of ssDNA. The signals of ssDNA and total DNA were quantified using imageJ software, and ssDNA signal was normalized to total DNA signal at each time point. The results shown were mean±S.E. of five similar experiments. * *p<0.05*; ** *p<0.01*. (**C**) MDA-MB-468 cells were transfected with a Cav-1-targeted siRNA or a non-targeting control (NT). Forty-eight hours later, the transfected cells were irradiated (5 Gy) for the indicated period of time followed by Western blot analysis of γ-H_2_AX. Levels of γ-H_2_AX and H_2_AX were quantified using imageJ software. γ-H_2_AX/H_2_AX ratios of untreated samples (zero time) were arbitrarily set at 100 as controls, and the treated samples were normalized to the controls. Results shown are the representative of three similar experiments; each point represents mean ± SD of triplicate determinations. * *p<0.05*, ** *p<0.01*.

To further define the roles Cav-1 in DNA damage response, we examined the effects of Cav-1 on signaling pathways that participate in DNA repair. siRNAs were utilized to inhibit Cav-1 expression. To avoid “off-target” effects of siRNA, we used two Cav-1-targeted siRNA sequences that both knocked down Cav-1 expression (Fig. 3A). We found that IR - induced accumulation of single-strand DNA (ssDNA) was increased in the cells transfected with the Cav-1-targeted siRNA, as compared to the cells transfected with a non - targeting RNA (Fig. 3B). The levels of γ-H2AX, the phosphorylated form of H2AX (at Ser^139^) associated with DSB, were also significantly higher in MDA-MB-468 cells with silencing of Cav-1 expression than in cells treated with a non-targeting RNA following IR (Fig. 3C), another evidence for defective DNA repair caused by loss of Cav-1. These observations suggest that Cav-1 defect may impair DNA damage repair.

**Figure 4:**
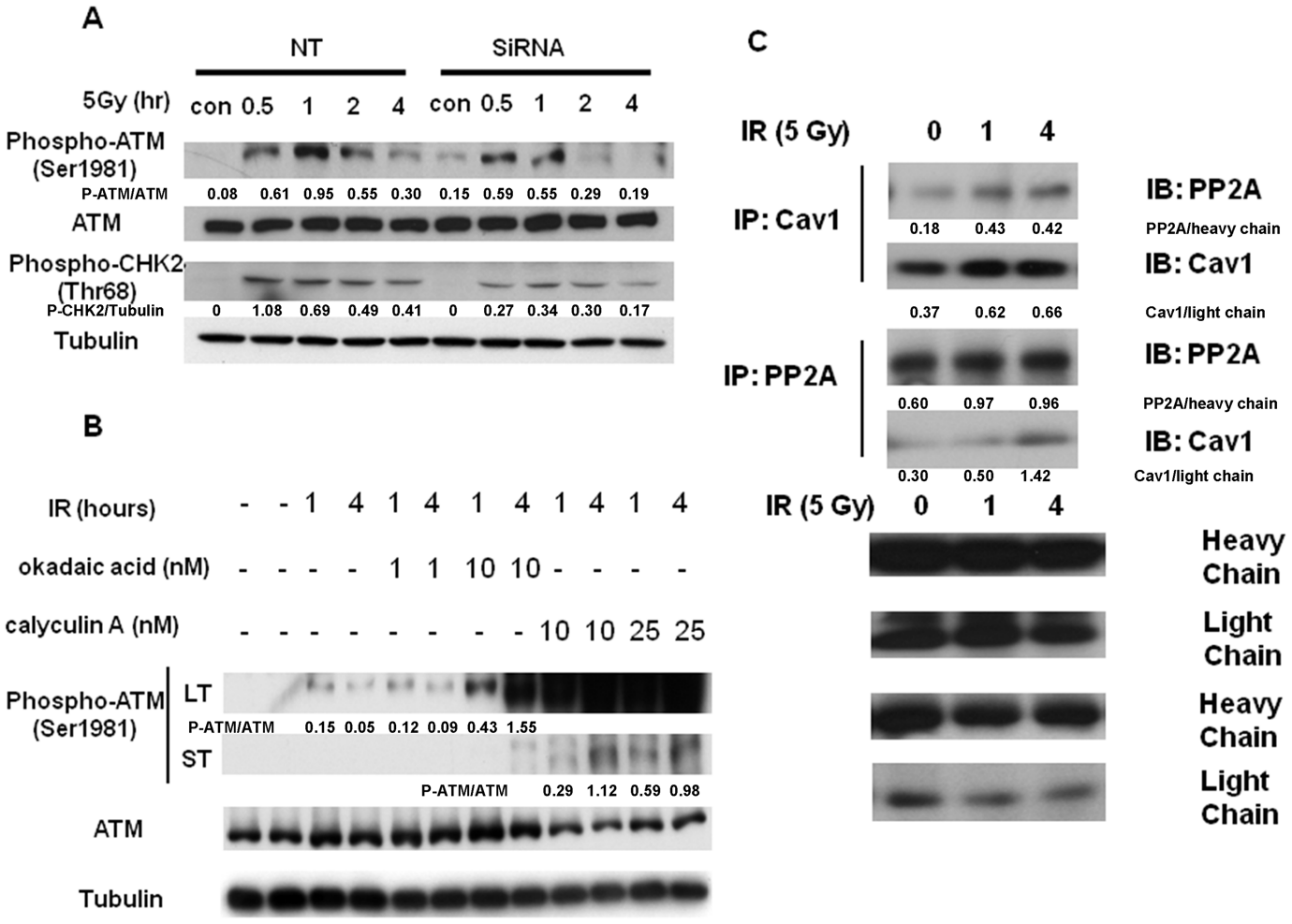
Cav-1-mediated inhibition of PP2A is responsible for the IR-induced accumulation of phospho-ATM. (**A**) MDA-MB-468 cells were transfected with a Cav-1 siRNA or a non-targeting RNA, followed by IR (5Gy) for the indicated period of time. The treated cells were collected for Western blot analysis of phospho-ATM, total ATM, phospho-CHK2, and tubulin. (**B**) MDA-MB-468 cells were irradiated (5Gy) for the indicated period of time in the absence or presence of the PP2A inhibitors, okadaic acid or calyculin A. The treated cells were collected for Western blot analysis of phospho-ATM and total ATM. Tubulin was used as a loading control. In order to show changes of phosphor-ATM, the results of two exposures were included. LT: 1 min exposure; ST: 10 sec exposure. (**C**) MDA-MB-468 cells were irradiated (5 Gy) for the indicated period of time, followed by immunoprecipitation and immunoblotting with the indicated antibodies. The results shown are the representative of three similar experiments.

**Figure 5:**
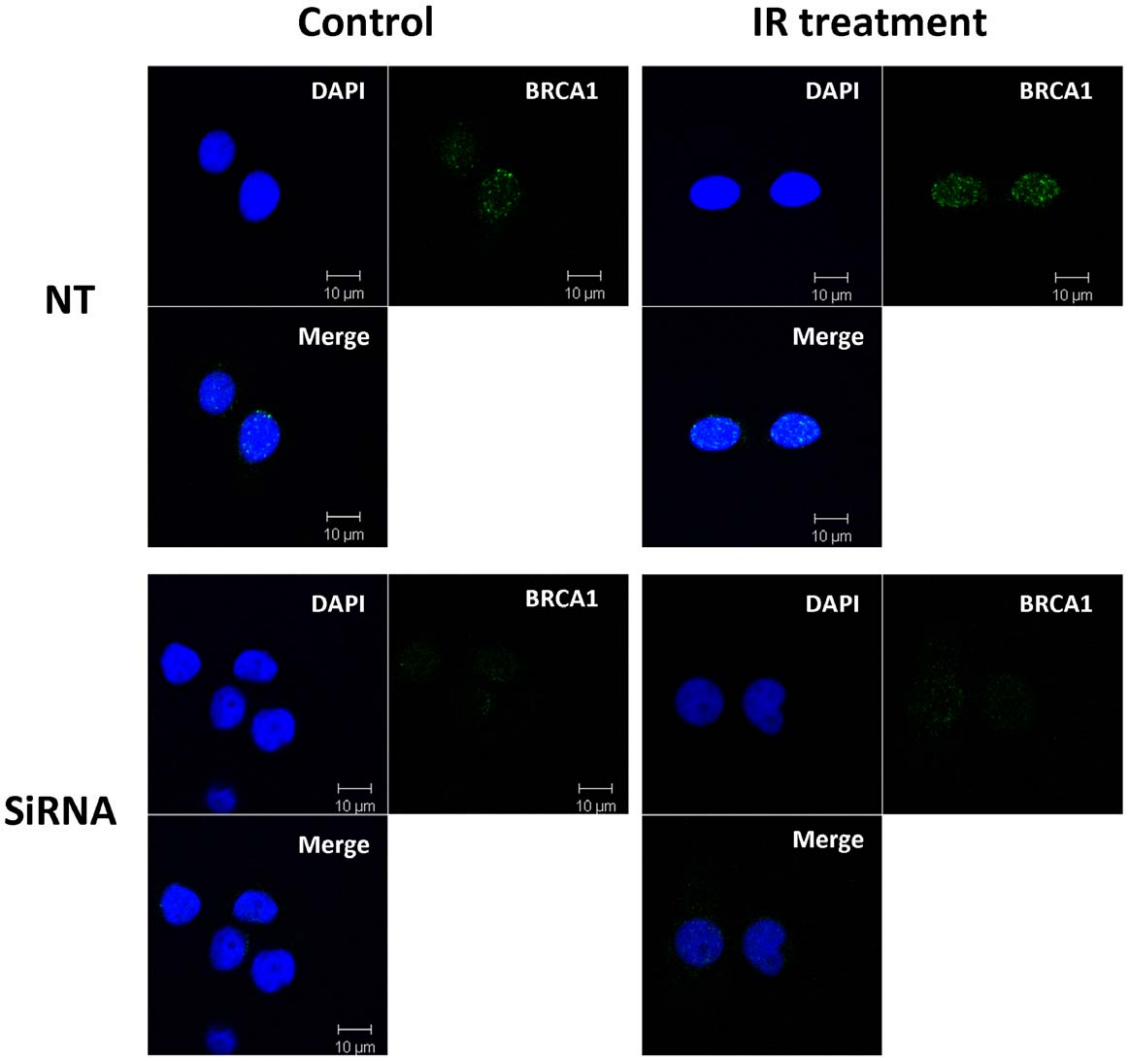
Silencing of Cav-1 expression decreases the IR-induced formation of BRCA1 foci. MDA-MB-468 cells were transfected with a Cav-1 siRNA or a non-targeting RNA. Forty-eight hours later, the cells were irradiated (5 Gy), and fixed for immunostaining with a BRCA1 antibody. BRCA1 foci were shown in green. DAPI was used for nucleus staining.

**Figure 6:**
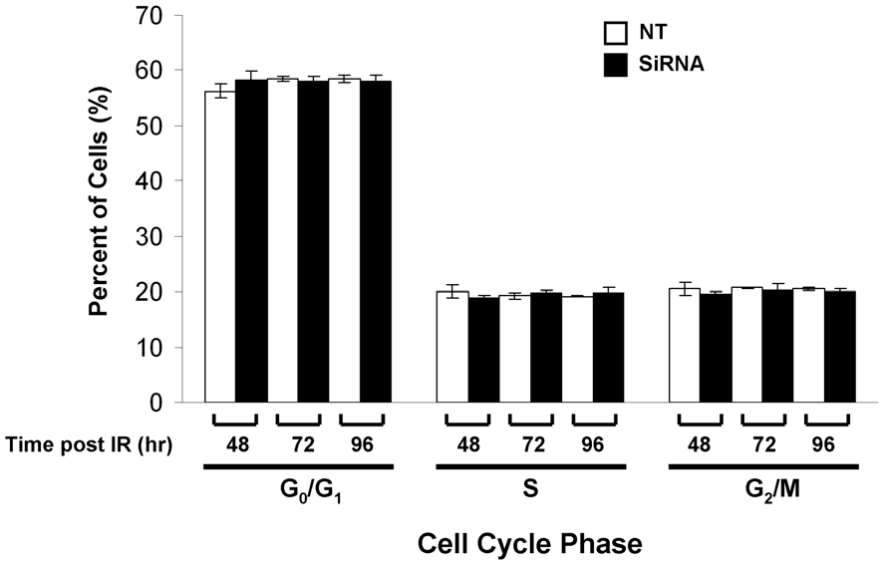
The effect of silencing of Cav-1 expression on cell cycle distribution. MDA-MB-468 cells with or without silencing of Cav-1 were fixed for cell cycle analysis by FACS at the indicated time following IR treatment. The results shown are the representative of three similar experiments; each bar represents the mean±S.D. of triplicate determinations.

Furthermore, we investigated whether suppression of Cav-1 resulted in impairment of DNA damage signaling. As shown in Fig. 4A, the activity of ATM, a kinase that is activated by DNA damage signals and phosphorylates a series of downstream targets such as CHK2, was lower in the cells with silencing of Cav-1 than that in the control cells following IR, as demonstrated by decreased levels of the phospho-ATM (Ser^1981^) and phospho-CHK2 (Thr^68^). Treatment of cells with inhibitors (okadaic acid and calyculin A) of PP2A, a phosphatase that decreases ATM phosphorylation, augmented the IR-induced phosphorylation of ATM (Fig. 4B), indicating the involvement of PP2A in the regulation of ATM activity in response to DNA damage. Moreover, our co-immunoprecipitation experiments demonstrated an increased physical association between Cav-1 with PP2A following IR (Fig. 4C). These results suggest that in response to DNA damage, Cav-1 plays an essential role in activating the ATM-initiated repair pathway by sequestering and inhibiting the function of PP2A. Also, using immunofluorescent microscopy we observed that knockdown of Cav-1 by siRNA reduced both the spontaneous and IR-induced foci formation of BRCA1, a DNA repair protein whose expression is controlled by Cav-1 (Fig. 5). The reduction of BRCA1 foci did not appear to be a consequence of changes in cell cycle, as the silencing of Cav-1 had no effect on cell cycle distribution (Fig. 6). These observations provide additional evidence that depletion of Cav-1 weakens the ability of cells to repair damaged DNA.

### Expression of Cav-1 is required for HR repair of damaged DNA

**Figure 7:**
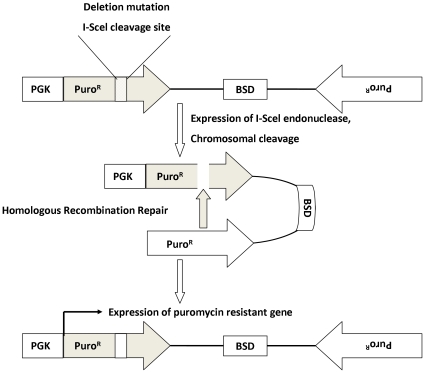
The schematic illustration of HR reporter assay.

**Figure 8:**
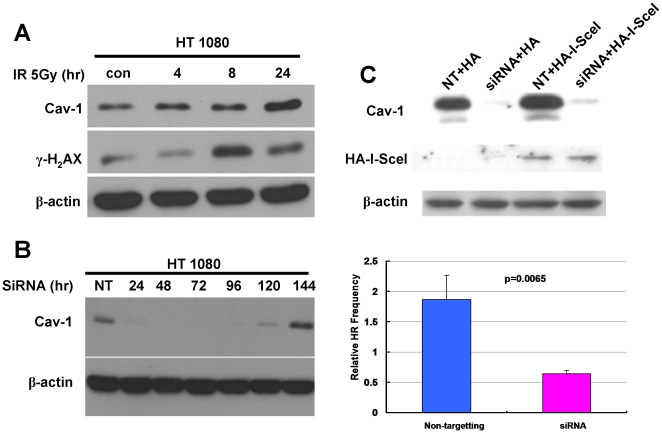
Silencing of Cav-1 expression reduces the DSB repair by HR. (**A**) HT-1080 cells were irradiated (5 Gy) for the indicated period of time, and then cell lysates were prepared for Western blot analysis of Cav-1 and γ-H_2_AX. β-actin was used as a loading control. (**B**) To determine the turnover of the silencing effect of Cav-1 siRNA in HT-1080 cells, we performed Western blot of analysis of Cav-1 at the indicated time after siRNA transfection. (**C**) HT-1080 cells were transfected with a non-targeting RNA or Cav-1 siRNA. Twenty-four hours after transfection, the cells were transfected with an HA tagged I-SceI endonuclease expressing vector (HA-I-SceI) or empty vector (HA) by electroporation, followed by Western blot analysis of Cav-1 and HA-I-SceI (upper panels), and by puromycin screening for HR frequency (lower panels). HR frequency was calculated as follows: the average numbers of colonies/dish were divided by the plating efficiency of transfection and divided by 85,000 (the total number of cells plated). The results shown are the representative of three similar experiments; each bar represents the mean±S.D. of triplicate determinations.

To begin to explore the mechanism by which Cav-1 regulates DNA repair, we first tested whether silencing of Cav-1 expression by siRNA altered the frequency of HR, one of the major pathways involved in DSB repair. We used HT1080 cell line and an HR reporter system developed by Brenneman et al [35]. HT1080 cell line carries a single integrated copy of a *puro* direct repeat HR substrate. One of the *puro* repeats is driven by the *PGK* promoter, but is inactive due to the insertion of an I-SceI recognition site; the second allele codes the wild-type protein, but lacks a promoter (Fig. 7). Introduction of an I-SceI expression vector into HT1080 cells creates DSBs at the I-SceI site, and only repair of these DSBs by HR can produce a functional *puro* that confers puromycin resistance. Fig. 8A demonstrates that similar to other Cav-1-expressing cell lines, HT1080 cells showed an increased expression of Cav-1 following IR. To determine the effect of Cav-1 on HR, HT1080 cells were transfected with an I-SceI expression vector, selected with puromycin, and then treated with a Cav-1 siRNA or non-targeting RNA. In HT1080 cells, the silencing effect of Cav-1 siRNA could last until day 6 after transfection (Fig. 8B), which is within the timeframe required assaying HR. Fig. 8C shows that there was an equal level of I-SceI expression in Cav-1 knockdown and control cells, but the Cav-1 knockdown cells had significantly lower level of HR after I-SceI expression.

### Expression of Cav-1 promotes NHEJ in response to DNA damage

**Figure 9:**
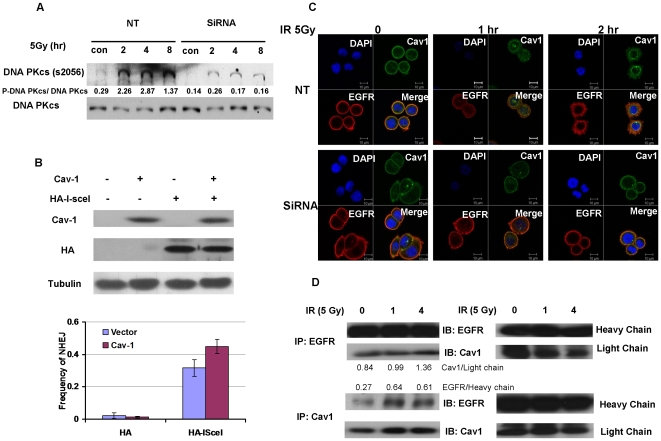
Expression of Cav-1 expression contributes to the activity of the NHEJ repair pathways. (**A**) MDA-MB-468 cells with or without silencing of Cav-1 were irradiated (5 Gy) for the indicated periods of time, and then subjected to Western blot analysis of phosphor-DNA-PKcs and total DNA-PKcs. (**B**) *Left panel*: HEK 293 cells containing a GFP-based chromosomal reporter, EJ5-GFP, were transfected with a caveolin-1 expression vector or a control empty vector. Thirty-six hours later, the cells were transfected with an HA tagged I-SceI endonuclease expression vector or an empty vector. Expressions of Cav-1 and HA-I-SceI were determined by Western blot. *Right panel*: Seventy-two hours following transfection with the HA-I-I-SceI plasmid, percentage of EGFP expressing cells, which represents the frequency of NHEJ, were determined by flow cytometry. The results shown are the mean ± S.E. from three identical experiments. (**C**) MDA-MB-468 cells with or without silencing of Cav-1were irradiated (5 Gy) for the indicated periods of time, and then fixed for immunostaining with Cav-1 and EGFR antibodies. Cav-1 staining was shown in green and EGFR staining in red. DAPI was used for nucleus staining. (**D**) MDA-MB-468 cells were irradiated (5 Gy) for the indicated periods of time. At the end of IR, cells lysates were prepared and subjected to immunoprecipitation and immunoblotting with either Cav-1 or EGFR antibodies as indicated. The light chain and heavy chains were used as loading controls.

NHEJ is another major pathway for mammalian cells to repair DSB [36]. As shown above in Fig. 6, silencing of Cav-1 reduced the foci formation of BRCA1. As BRCA1 is a protein know to be involved in DSB signal transduction and may regulate both HR and NHEJ, we next wanted to know if Cav-1 is involved in the regulation of this important DNA repair pathway. The phosphorylation of DNA-PK, one of the necessary components of the NHEJ pathway, was used as a read-out of this repair system. We found that although exposure of MDA-MB-468 cells to IR markedly induced DNA-PK phosphorylation at Ser^2056^, suppression of Cav-1 expression by siRNA effectively inhibited the IR- stimulated phosphorylation of this critical DSB repair factor (Fig. 9A), suggesting that Cav-1 is involved in controlling the activity of the NHEJ pathway. The results of the NHEJ assay, which measures overall frequency of NHEJ [37], demonstrated that introduction of Cav-1 into HEK293 cells (Fig. 9B, left panel) significantly increased the NHEJ frequency as compared to the transfection with a control empty vector (a 40% increase was observed) (Fig. 9B, right panel). To further analyze how Cav-1 regulates the phosphorylation of DNA-PK, we examined the effect of Cav-1 on IR-induced nuclear translocation of EGFR, which is known to interact with DNA-PK and promote its phosphorylation [38]. To further examine the localization of Cav-1 and EGFR in response to IR treatment, we immunostained the cells with Cav-1 (in green) and EGFR (in red) before and after IR treatment. As shown in Fig. 9C, Cav-1 and EGFR were located on plasma membrane before IR treatment, but co-translocations of Cav-1 and EGFR in the nuclei were observed 1 h following IR, as visualized by confocal microscopy. By contrast, the nuclear co-translocation of these two proteins was barely seen in the cells with silencing of Cav-1 (Fig. 9C). The similar intensity of Cav-1 staining in the cells transfected with a Cav-1 siRNA or a non-targeting RNA was likely due to the high affinity of the Cav-1 antibody and the high sensitivity of the immunofluorescence detection method. Physical association between Cav-1 and EGFR also increased following IR (Fig. 9D). These results suggest that Cav-1 can control NHEJ through modulating the activity of DNA-PK via Cav-1-mediated nuclear translocation of EGFR.

### Silencing of Cav-1 expression increases sensitivity of cancer cells to genotoxic stresses

**Figure 10:**
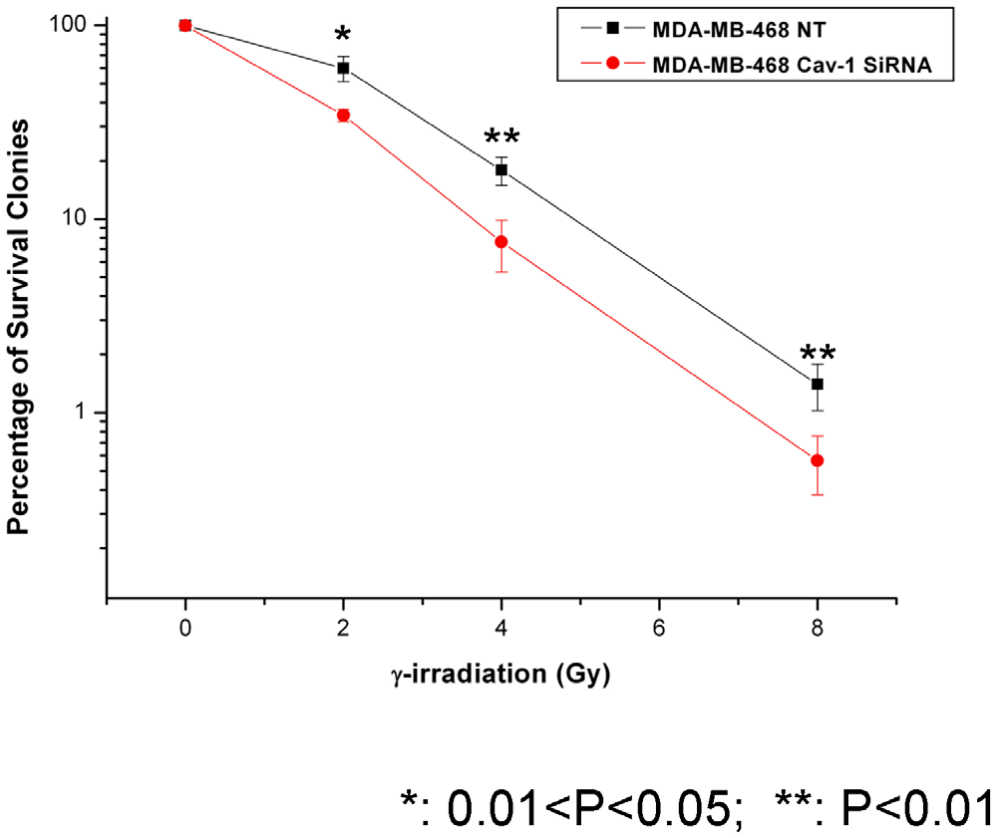
Silencing of Cav-1 expression sensitizes cells to IR and bleomycin. MDA-MB-468 cells with or without silencing of Cav-1 were treated with varying doses of γ-radiation, and colony formation assay was performed to compare cell survival. Results shown are the representative of three similar experiments; each point represents mean ± SD of quadruplicate determinations of the experiment.

To assess the functional significance of the up-regulation of Cav-1 in response to DNA damage, we examined the effect of silencing of Cav-1 expression on survival of the cells treated with IR, using a colony formation assay. Fig. 10 shows that IR caused a significantly more killing in the cells with loss of Cav-1 than in the control cells, further supporting a role of Cav-1 in protecting cells against genotoxic stress.

## Discussion

Loss of the putative tumor suppressor, Cav-1, is believed to be one of the causes for development of several types of cancers, but evidence also show that overexpression or re-expression of Cav-1 in advanced stages of the disease may contribute to tumor progression. Yet, how loss of Cav-1 facilitates tumorigenesis and how re-induction of Cav-1 promotes tumor progression remain an open question. It has been reported that expression of Cav-1 favors cancer cell proliferation by regulating survival pathways such as Rac, Erk and PtdIns 3-kinase [39] and inhibits detachment-induced apoptosis (anoikis) either through suppressing p53 activation [40] or up-regulating the transcription of the IGF-I receptor gene [41]. We previously demonstrated that Cav-1- regulated calcium homeostasis plays a role in growth and survival of breast cancer cells [42]. In the present study, we sought to determine the functional significance of Cav-1 up-regulation caused by treatments with DNA damaging agents, a phenomenon that was also observed by others [33], [34], [43]. Our study reveals a new function of Cav-1 as a possible sensor and mediator in the DNA damage response/repair process. We show that expression of Cav-1 can be rapidly up-regulated by DNA damaging agents such as IR (Fig. 1), and that the up-regulation of Cav-1 protein plays a critical role in activating the DNA repair signaling cascade, since depletion of Cav-1 expression by siRNA impairs the cells' ability to repair DNA, as evidenced by increased accumulation of g-H_2_AX (Fig. 3C) and ssDNA (Fig. 3B), reduced phosphorylation of ATM at Ser^1981^ and CHK2 at Thr^68^ (Fig. 4A), and decreased formation of BRCA1 foci (Fig. 5). Moreover, our study reveals for the first time that Cav-1 is able to regulate both HR and NHEJ pathways, two major mechanisms responsible for repair of DNA DSB. This conclusion is supported by use of two assays specific for detecting HR and NHEJ. In the current study, the repair of DSBs induced by the I-SceI endonuclease is monitored using artificial chromosome-integrated reporters, namely HT1080-1885 for HR pathway (Fig. 8) and EJ5-GFP for NHEJ pathway (Fig. 9). Each individual reporter is designed such that repair of I-SceI-induced DSBs by a specific pathway restores a puromycin resistance or a GFP expression cassette. In each of the reporter-containing cell lines, the activation of the reporter is confirmed to be dependent upon expression of I-SceI. The restoration of puromycin resistance in HT1080-1885 can only be achieved by HR repair of I-SceI induced DSB using downstream homologue as template. For EJ5-GFP cells, a promoter is separated from a GFP coding cassette by a puro gene that is flanked by two I-SceI sites in the same orientation. Once the puro gene is excised by NHEJ repair of the two I-SceI-induced DSBs, the promoter is joined to the rest of the expression cassette, leading to restoration of the GFP+ gene.

We demonstrated that IR induced the expression of Cav-1 (Fig. 1), a phenomenon previously reported by others [33], [34], [43], (Fig. 1), but we also found that the increased expression of Cav-1 protein by IR does not appear to result from activation of *Cav-*1 transcription, as the mRNA level of Cav-1 was not affected by the treatments (Fig. 2). The exact mechanism in which Cav-1 increased after DNA damage remains to be elucidated. Our observation on the roles of Cav-1 in activating DNA repair signaling (Fig. 3, 4, and 5) may explain the pro-survival function of Cav-1 in IR-treated cells, as shown by us (Fig. 10) and others [33], [43], [44]. Notably, we found that Cav-1 could be up-regulated within 30 min following IR treatment (Fig. 1), earlier than the 24 h shown by Cordes et al [33], suggesting that Cav-1 may act as a sensor and early mediator in response to DNA damage.

In this study we have begun to elucidate the mechanisms by which Cav-1 regulates DNA repair. We demonstrated that Cav-1 participates in both HR and NHEJ repair pathways. The effect of Cav-1 on HR was demonstrated by the experiments showing that silencing of Cav-1 expression decreased HR frequency (Fig. 8). The role of Cav-1 in HR might be related to, at least in part, its effect on the accumulation of BRCA1 foci in nuclei after DNA damage (Fig. 5), which was verified by cell cycle analysis showing that knockdown of Cav-1 did not alter cell cycle distribution, a factor known to affect the foci formation of BRCA1 protein [45]. Reciprocal regulation of the expression of Cav-1 and BRCA1 has been reported [46], [47], but whether this is associated with the Cav-1-mediated BRCA1 nuclear accumulation remains to be clarified. The role of Cav-1 in NHEJ was supported by our observation that suppression of Cav-1 by siRNA dramatically inhibited the IR-activated phosphorylation (Ser2056) of DNA-PK (Fig. 9A), one of the key executers in the NHEJ system, and by the GFP-based chromosomal reporter assay showing that the frequency of NHEJ was significantly higher in HEK293 cells transfected with a Cav-1 expression vector than in the cells transfected with a control vector (Fig. 9B). The mechanism of these effects might involve the Cav-1-mediated nuclear translocation of EGFR, an activator of DNA-PK [43], as Cav-1-targeted siRNA also inhibited the co-translocation of Cav-1 and EGFR following IR treatment (Fig. 9C and D). Therefore, it is likely that the signaling – modulating molecule, Cav-1, may facilitate DNA repair via multiple pathways. How precisely Cav-1 regulates HR and NHEJ and whether Cav-1 is involved in other DNA repair pathways remain to be studied.

Our results may provide a possible explanation for the differential expression of Cav-1 at various stages of tumor progression. As genome instability triggered by endogenous or exogenous DNA damaging agents is one of the main causes of cancer, loss or deficiency of Cav-1 at early stages of cancer development may cause a defect in DNA damage response leading to genomic alteration and oncogenic transformation. However, re-expression of Cav-1 at later stages of cancer may provide a protective mechanism for cancer cells to survive various harsh conditions such as DNA damage. In fact, the protective effects of Cav-1 against mechanical shearing damage, hypoxia, and nutrient depletion, the stresses that are considered the causes for death of tumor cells during their migration and metastasis, have been reported recently [48], [49], [50], [51], [52]. Thus, re-expression of Cav-1 at advanced stages of cancer may provide a survival mechanism for tumor cells, and targeting Cav-1 may represent a new stratagem for cancer treatment.

Taken together, our study reveals a novel function for Cav-1 in repairing DNA, which involves both HR and NHEJ, and suggests that Cav-1 may play a critical role in orchestrating the response of cells to DNA damage and in mediating DNA repair.

## Materials and Methods

### Cell culture

MDA-MB-468 (human breast cancer cell), MCF-7 (human breast cancer cell), MCF-10A (human mammary epithelial cell), PC-3 (human prostate cancer cell) and T98G (human glioma cell) lines were purchased from American Type Culture Collection (Rockville, MD). NCI/ADR-RES line (ovarian cancer cell line) (previously named MCF-7/AdrR) was provided by Dr. Kenneth Cowan of the Eppley Institute for Research in Cancer (Omaha, NE). HT1080 (human fibrosarcoma cell) line was obtained from Dr. Mark Brenneman (Rutgers University, Piscataway, NJ). MCF-7, PC-3 and NCI/ADR-RES cell lines were maintained in RPMI 1640 medium (Invitrogen Life Technologies, Gaithersburg, MD); MDA-MB-468 and HT1080 cell lines in Dulbecco's modified Eagle's medium (Invitrogen Life Technologies); T98G in Ham's F-10/DMEM (10∶1) medium (Invitrogen Life Technologies); and MCF-10A in DMEM/F12 (Invitrogen Life Technologies) supplemented with 5% donor horse serum, 20 ng/ml epidermal growth factor, 10 µg/ml insulin, 0.5 µg/ml hydrocortisone and 100 ng/ml cholera toxin (Sigma, St. Louis, MO). All the culture media contained 100 units/ml penicillin and 100 µg/ml streptomycin (Invitrogen Life Technologies, Gaithersburg, MD); all the cell lines were cultured and grown in a 5% CO_2_ - humidified incubator at 37°C.

### siRNA transfection

Cells in exponential phase of growth were plated in 60-mm cell culture plates at 1×10^6^ cells/plate and incubated for overnight, and then transfected with 100 nM of Cav-1 siRNA or a non-target RNA (Dharmacon, Inc, Lafayette, CO) using Lipofectamine 2000 and OPTI-MEM I reduced serum medium (Invitrogen Life Technologies, Gaithersburg, MD), according to the manufacturer's protocol. Silencing effects of siRNA were examined by Western blot and real-time RT-PCR.

### Western blot analysis

Cells were washed twice with PBS containing a Protease Inhibitor Cocktail (Pierce Biotechnology Inc., Rockford, IL) and lysed with CelLytic™ MT Cell Lysis Reagent (Sigma-Aldrich, St. Louis, MO). Lysates were transferred to 1.5-ml eppendorf tubes and clarified by centrifugation at 16,000×g for 25 min at 4°C. Equal amounts of cell lysates (25 µg proteins) were resolved by SDS-PAGE, and then transferred to nitrocellulose. The membranes were incubated in 5% nonfat milk in TBST (Tris-buffered saline plus 0.1% Tween 20) at room temperature for 1 h, followed by immunoblotting with the respective antibodies. Detection of proteins by enzymed-linked chemiluminescence was performed according to the manufacturer's protocol (ECL; Pierce Biotechnology Inc., Rockford, IL). Quantification of protein bands was performed using the ImageJ software (http://rsb.info.nih.gov/ij). The antibodies used and dilution ratio were: mouse anti-β-actin (AC-74), anti-tubulin (DM1A) antibodies (1∶5000; Sigma-Aldrich, St. Louis, MO); mouse anti-BRCA1(AB-1) antibody (1∶500; Calbiochem, La Jolla, CA); mouse anti-Cav-1 (Z034) (1∶2000; Zymed Laboratories, San Francisco, CA, USA); rabbit anti-EGFR (1∶1000; Cat. No. SC-03, Santa Cruz Biotech, Santa Cruz, CA); rabbit anti-H_2_AX (ab2893), anti-DNA PKcs (ab32566) and anti-DNA PKcs (phospho S2056) (ab18192) antibodies (1∶2000, 1∶1000 and 1∶500; Abcam, Cambridge, UK); mouse anti-H_2_AX (phospho S139) (JBW301) antibody (1∶2000; Upstate, Chicago, IL); mouse anti-PP2A-C (1D6) antibody (1∶1000; Chemicon International, Chandlers Ford, UK); rabbit anti-ATM (D2E2), anti-ATM (phospho S1981) (10H11.E12)and anti-CHK2 (phospho T68) (C13C1) (1∶2000, 1∶1000 and 1∶500; Cell Signaling Technology, Beverly, MA).

### Quantitative real-time RT-PCR

Total RNAs from cells were extracted with TriZol Reagent (Invitrogen Life Technologies, Gaithersburg, MD) following the manufacturer's instruction. First strand cDNA synthesis and amplification were performed using Omniscript RT Kit (QIAGEN Valencia, CA). The following human *CAV1* primers were used: forward: 5′-CAC ATC TGG GCA GTT GTA CC-3′; reverse: 5′-CAC AGA CGG TGT GGA CGT AG-3′ [53]. The *β-actin* primers, designed by our laboratory [54], were as follows: forward: 5′-GCC AAC ACA GTG CTG TCT GG-3′; reverse 5′-GCT CAG GAG GAG CAA TGA TCT TG-3′. SYBR Green quantitative PCR amplifications were performed on the Stratagene 3005P Real-TimePCR system. Reactions were carried out in a 25-µl volume containing 12.5 µl of 2 SYBR Green PCR Master Mix (Bio-Rad). The thermal profile for the real-time PCR was 95°C for 3 min followed by 40 cycles of 95°C for 20 s, 59°C for 30 s, and 70°C for 30 s. The ΔC_t_ data were collected automatically. The average ΔCt of each group was calculated by the following formula: ΔC_t_ = average *CAV1* gene C_t_-average of HK (housekeeping) gene' Ct. ΔΔCt was calculated by ΔΔCt = ΔC_t_ of non-target control group - ΔC_t_ of the siRNA transfection group. The fold-change for *CAV1* expression level was calculated using 2^−ΔΔCt^.

### Immunofluorescence

Cells were cultured on glass slides and stained as described previously [55]. Briefly, cells were treated with 5 Gy of IR, and then fixed in PBS containing 4% paraformaldehyde (EMD Chemicals, Merck Corporation, San Diego, CA) at room temperature for 20 min. Fixed cells were rinsed with PBS and with 25 mM NH_4_Cl in PBS for 10 min to quench free aldehyde groups. The cells were permeabilized by incubation in freshly-prepared 0.1% Triton X-100/PBS for 15 min. The cells were pre-incubated for 1 h in PBS containing 3% bovine serum albumin (BSA) and incubated for 2 h in diluted antibodies in PBS containing 3% BSA. Following washing three times, the cells were incubated for 1 h in diluted flourescence–labeled secondary antibodies. After washing with PBS, immuno-stained cells were examined with a Zeiss LSM 510 Meta laser scanning confocal microscope or ApoTome Microscope (Carl Zeiss Ltd, Germany). Primay antibodies and dilution ratio were: rabbit anti-Cav-1 antibody (1∶1000; ab2910, Abcam, Cambridge, UK); mouse anti-BRCA1 antibody (1∶50; Calbiochem, La Jolla, CA); sheep anti-EGFR (1E4) antibody (1∶400; Upstate, Chicago, IL); mouse anti-PP2A-C antibody (1∶100; Chemicon International, Chandlers Ford, UK); mouse anti-H_2_AX (phospho S139) antibody (1∶2000; Upstate, Chicago, IL). All secondary antibodies were purchased from Molecular Probes (Eugene, OR) and used at a dilution of 1∶500.

### Immunoprecipitation

Cells were washed with PBS, collected, and lysed with RIPA buffer (150 mM NaCl, 1% NP-40, 0.5% deoxycholate, 0.1% SDS, 50 mM Tris pH 8.0) containing Protease Inhibitor Cocktail (Pierce Biotechnology Inc., Rockford, IL). The lysates were sonicated and centrifuged at 16,000×g for 25 min at 4°C. The supernatant was pre-cleared with protein A/G agarose (1∶25 dilution), and immunoprecipitation was carried out using the respective antibodies. The immuno-complexes were washed four times with RIPA buffer, and proteins were eluted with 2X SDS sample buffer by boiling for 5 min. Ten µl precipitated proteins were resolved on SDS–PAGE and subjected to Western blot analysis using the respective antibodies.

### Immunofluorescent detection of ssDNA

Detection of ssDNA was carried out as reported [56]. Briefly, cells were grown on cover slips for overnight, and then incubated in medium containing 30 mmol/l BrdU (Bromodeoxyuridien, Sigma) for 24 h in the dark. To visualize ssDNA, the cells were fixed with methanol at −20°C for 10 min and then incubated in blocking solution (2% bovine serum albumin in PBS) at room temperature for 30 min, followed by incubation with an anti-BrdU antibody (Becton Dickinson, Franklin Lakes, NJ, USA). The cells were washed four times with PBS and then incubated with rhodamine red-X- conjugated goat anti-mouse IgG (Jackson ImmunoResearch Laboratories, Inc.) for 30 min at room temperature in the dark. The cells were counterstained with DAPI in blue for total DNA. Fluorescent images were taken using Carl Zeiss fluorescent microscope (Axiovert-200M) equipped with a Carl Zeiss digital camera (AxioCam MRC). The nuclear areas were selected using the ImageJ v1.37 software (http://rsb.info.nih.gov/ij/) and fluorescent signals from ssDNA and total DNA were integrated. The ratio of ssDNA signal intensity to total DNA represents the relative level of ssDNA in the nucleus. For each experiment, at least 30 cells were analyzed. Data were reported as mean ± SE from five independent experiments. Statistical significance of fluorescent signal intensity and ratio of signal intensity in cells with or without silencing of Cav-1 were analyzed by two-tailed t-test.

### HR assay

Human HT1080 cells were transfected with either a Cav-1 siRNA or a non-targeting RNA. Two days after transfection, cells were trypsinized and resuspended in gene pulser electroporation buffer. Four µg of an I-SceI expression vector pCMV(3_NLS) HA-I-SceI or an empty vector were introduced into 3.5×10^5^ cells by electroporation. The cells were then seeded at 85,000 cells/100-mm culture dish for puromycin selection. The seeded cell cultures were re-fed with fresh medium containing 1 µg/ml puromycin on day 2 following electroporation, and the puromycin - containing medium was changed on days 6, 10, 12, and 14 days. At the end of selection, cells were fixed and stained, and colonies with 50 or more cells were counted.

### NHEJ frequency assay

The frequency of total NHEJ was determined using a GFP-based chromosomal reporter, EJ5-GFP, as previously described by Bennardo et al. [37]. Briefly, the transformed human embryonic kidney HEK293 cells containing a GFP-based chromosomal reporter, EJ5-GFP, were transfected with a caveolin-1 expression vector or a control empty vector. Thirty-six hours later, the cells were transfected with an HA tagged I-SceI endonuclease expression vector or a control empty vector. Seventy-two hours following transfection with the HA-I-I-SceI plasmid, percentage of EGFP expressing cells, which represents the frequency of NHEJ, were determined by flow cytometry.

### Analysis of cell cycle distribution

Cells were trypsinized, washed, and fixed with 80% ethanol for 1 hour. Following treatment of cells with RNase and propidium iodide (Sigma, St. Louis, MO), cell cycle distribution (5,000 cells) was analyzed by fluorescence activated cell sorting (FACS) using a flow cytometer (Coulter Cytomics FC, Beckman Coulter, Miami, FL).

### Colony formation assay

The numbers of cells to be plated for each IR dose and drug concentration were determined by a pilot experiment in order to yield 50–150 surviving colonies/100-mm plate. For IR tests, cells were plated and incubated for 18 h at 37°C, and then were irradiated with a Cs-137 γ-irradiator (Nordion Inc., Canada). Two weeks after IR treatment, colonies were fixed with methanol and stained with 1% crystal violet. To calculate the survival fraction, number of colonies was normalized to the number of cells plated. For drug tests, cells in 60 - mm plates were treated with bleomycin (EMD Chemicals, Merck Corporation, San Diego, CA) for 2 h, and then the drug was washed off with drug-free medium. The survival fractions were calculated as described above.

### Statistical analysis

Student's *t*-test was used to determine the degree of significance.
